# Anticancer effects of SH003 and its active component Cucurbitacin D on oral cancer cell lines via modulation of EMT and cell viability

**DOI:** 10.32604/or.2025.059791

**Published:** 2025-04-18

**Authors:** HYEONG SIM CHOI, JEONG-KUI KU, SEONG-GYU KO, PIL-YOUNG YUN

**Affiliations:** 1 Department of Oral and Maxillofacial Surgery, Section of Dentistry, Seoul National University Bundang Hospital, Seongnam, 13620, Republic of Korea; 2 Department of Preventive Medicine, College of Korean Medicine, Kyung Hee University, Seoul, 02435, Republic of Korea; 3 Department of Dentistry and Dental Research Institute, School of Dentistry, Seoul National University, Seoul, 03080, Republic of Korea

**Keywords:** SH003, Oral cancer, Cucurbitacin D, Epithelial–mesenchymal transition (EMT), Migration

## Abstract

**Background:**

Oral cancer remains a significant global health challenge, as it has high morbidity and mortality rates. Current treatments show limited efficacy and have severe side effects, prompting searches for new therapeutic agents. SH003, a traditional herbal formulation comprising *Astragalus membranaceus*, *Angelica gigas*, and *Trichosanthes kirilowii*, has demonstrated potential anticancer properties in previous studies. However, its specific efficacy against oral cancer and the role of its key components, particularly Cucurbitacin D, remain underexplored.

**Methods:**

The cytotoxic effects of SH003 and its major components—i.e., Cucurbitacin D, Decursin, Formononetin, and Nodakenin—were evaluated using 3-(4,5-Dimethylthiazol-2-yl)-2,5-Diphenyltetrazolium Bromide (MTT), Trypan Blue exclusion, and Lactate Dehydrogenase (LDH) release assays. Cell migration was analyzed via wound healing assays, and apoptosis induction was assessed using cell cycle analysis and caspase activation assays. Epithelial-to-mesenchymal transition (EMT) marker expression (E-cadherin and N-cadherin) was measured using Western blotting and Quantitative reverse transcription PCR (qRT-PCR).

**Results:**

SH003 significantly reduced cell viability in a dose-dependent manner, with YD-8 and YD-9 cells showing greater sensitivity than YD-38 cells. Of the individual compounds, Cucurbitacin D was identified as a key active agent, as it exhibited potent inhibition of cell migration and significant modulation of EMT markers, including the upregulation of E-cadherin and downregulation of N-cadherin. These effects were most pronounced in YD-9 cells.

**Conclusions:**

Taken together, these findings suggest that Cucurbitacin D plays a crucial role mediating the anticancer activity of SH003, particularly via the reversal of EMT and the reduction of migratory and invasive potential of oral cancer cells. This study provides valuable insight into the mechanistic basis of SH003, highlighting its potential as a therapeutic agent against oral cancer. Further research, including *in vivo* studies and clinical trials, is needed to elucidate its precise mechanisms and potential applications against other cancer types.

## Introduction

Oral cancer remains a significant global health challenge, with an estimated 389,485 new cases and 188,230 deaths reported worldwide in 2022 [1]. Despite advances in treatment—including surgery, radiation, chemotherapy, and targeted therapies—oral cancer continues to contribute to high morbidity and mortality rates, imposing substantial social and economic burdens on both patients and healthcare systems [2–4]. Current treatments often result in severe side effects, such as mucositis, xerostomia, and loss of taste, and are associated with challenges like recurrence and drug resistance, underscoring the urgent need for novel, more effective, and safer therapeutic agents [3,5].

Natural compounds have emerged as promising alternative to conventional treatments due to their ability to modulate key cancer-related processes, such as epithelial-mesenchymal transition (EMT) and cancer cell migration [6]. For instance, curcumin has been reported to Suppress EMT in oral cancer, while resveratrol has demonstrated anti-migratory effects in various cancer types [7–9]. Among natural compounds, herbal extracts stand out for their multifaceted biological activities, as they often combine anti-inflammatory, antiproliferative, and immunomodulatory effects [5,10]. SH003, a traditional herbal formulation containing *Astragalus membranaceus*, *Angelica gigas*, and *Trichosanthes kirilowii*, has shown promising anticancer properties in previous studies and is currently undergoing clinical trials, supporting its potential as a therapeutic agent [11,12].

While the individual components of SH003—Cucurbitacin D, Decursin, Formononetin, and Nodakenin—have demonstrated distinct anticancer effects, their potential synergistic interactions within SH003 remain poorly understood, particularly in the context of oral cancer [13–16]. For example, Cucurbitacin D targets various signaling pathways involved in cancer cell survival, while Decursin inhibits angiogenesis and metastasis. Formononetin induces apoptosis and cell cycle arrest, and Nodakenin exerts antiproliferative effects. The combination of these compounds may produce a synergistic effect, enhancing their individual anticancer activities to achieve a more potent therapeutic response.

This study evaluates the cytotoxic, antimigratory, and EMT-modulating effects of SH003 and its major components on oral squamous cell carcinoma (OSCC) cell lines YD-8, YD-9, and YD-38, which represent different stages and characteristics of oral cancer. Understanding SH003’s mechanisms of action could facilitate the identification of novel therapeutic targets and the development of combination therapies with existing anticancer agents. By addressing the critical gaps in our knowledge of SH003’s efficacy and mechanism, this research aims to contribute to the broader field of cancer therapy by highlighting the potential of natural compounds in developing safer and more effective treatments.

## Materials and Methods

### Cell lines and culture conditions

We obtained the human oral cancer cell lines YD-8, YD-9, and YD-38 from the Korean Cell Line Bank (KCLB, Seoul, Republic of Korea). These cell lines were selected due to their distinct p53 mutation statuses, representative of the diverse genetic profiles found in oral squamous cell carcinomas (OSCC). Specifically, YD-8 has point mutations at codon189 of exon 6 and 273 of exon 8 in the p53 gene, YD-9 expresses wild-type p53, and YD-38 has a point mutation at codon 282 and a p53 deletion of exon 8 [17]. All cells were cultured in RPMI-1640 medium (Biowest, L0496-500, Nuaillé, Maine-et-Loire, France) supplemented with 10% heat-inactivated fetal bovine serum (Biowest, S1480 (159A17), Nuaillé, Maine-et-Loire, France), 1% penicillin-streptomycin (Biowest, L0022-100, Nuaillé, Maine-et-Loire, France), and cultures were maintained in a humidified incubator at 37°C with 5% CO_2_. Although regular Mycoplasma testing is recommended for cell culture experiments, Mycoplasma contamination testing was not performed on these cell lines in our laboratory during this study. This limitation should be taken into account when interpreting the results.

### Preparation of SH003 and its components

SH003 is composed of *A. membranaceus*, *A. gigas*, and *T. kirilowii* in a 1:1:1 ratio. This mixture was provided by Hanpoong Pharm and Foods Company (Jeonju, Republic of Korea), which performed the extraction under Good Manufacturing Practive (GMP) conditions. The dried extracts were then dissolved in 30% ethanol to create a stock solution of 20 mg/mL, which was stored at −80°C until use. Standardization and quality control of SH003 were performed as previously described [18].

The major components of SH003—Cucurbitacin D (CID: 5281318; PubChem, Bethesda, MD, USA), Decursin (CID: 442126), Formononetin (CID: 5280378), and Nodakenin (CID: 73191)—were purchased from ChemFaces (Wuhan, China). Stock solutions of these compounds were prepared in dimethyl sulfoxide (DMSO) before being diluted to their desired concentration with culture medium before use.

The doses of SH003 and its components were selected based on previous studies and preliminary experiments. Earlier research demonstrated significant anti-cancer effects of SH003 at 0–500 μg/mL; thus, for this study, we applied a similar range, referencing studies on comparable concentrations in other cancer types. The doses for each individual components—Cucurbitacin D, Decursin, Formononetin, and Nodakenin—were also determined based on prior findings, ensuring that all doses remained below cytotoxic thresholds for oral cancer cells [18,19].

### Cell viability assays

We assessed cell viability using the MTT assay. This colorimetric assay measures cell metabolic activity as an indicator of cell viability. It’s widely used in cancer research to assess cell proliferation and the cytotoxic effects of drugs. Briefly, cells were seeded into 96-well plates at a density of 5 × 10^3^ cells/well before being allowed to adhere overnight. Cells were then treated with various concentrations of SH003 (i.e., 0, 100, 200, or 500 μg/mL) or its components (Cucurbitacin D: 0, 0.1, 0.2, or 0.5 μM; Decursin: 0, 25, 50, or 100 μM; Formononetin: 0, 25, 50, or 100 μM; Nodakenin: 0, 25, 50, or 100 μM) for 72 h. After this treatment, 20 μL of 3-(4,5-Dimethylthiazol-2-yl)-2,5-diphenyltetrazolium (MTT; Sigma, M2128-1G, St. Louis, MO, USA) solution was added to each well and the resulting mixture was incubated for an additional 4 h. The formazan crystals that were formed were then dissolved in 100 μL of DMSO (Samchun Pure Chemical, D0457, Pyeongtaek, Republic of Korea), and the absorbance of this solution was measured at 570 nm using a SpectraMax Plus 384 microplate reader (Molecular Devices, LLC, San Jose, CA, USA). All results were expressed as a percentage of cell viability relative to an untreated control.

### Trypan blue exclusion assays

Cytotoxicity and live/dead cell numbers were determined using Trypan Blue exclusion assays. It provides a straightforward measure of cell viability in cancer studies. To do so, we first seeded aliquots of 2.5 × 10^4^ cells to 48-well plates, then treated them with SH003 or its components. After these treatments, cells were harvested and stained with Trypan Blue dye (0.4% solution, Invitrogen, T10282, Grand Island, NY, USA). After staining, the number of viable (unstained) and nonviable (stained) cells were counted using a hemocytometer under an Olympus CKX53 inverted microscope (Olympus Corporation, Shinjuku, Tokyo, Japan). The percentage of viable cells was calculated as [(number of viable cells/total cells) × 100].

### LDH release assays

Next, cell membrane integrity was assessed using a CytoTox 96 Non-Radioactive Cytotoxicity Assay Kit (Promega, G1780, Madison, WI, USA). This assay measures the release of lactate dehydrogenase (LDH) from damaged cells into the culture medium, assessing cytotoxicity. It’s useful in cancer research for evaluating the cytotoxic effects of potential anti-cancer agents. For this assay, 2.5 × 104 cells were seeded into 48-well plates and treated with SH003 or its components for 15 h. After treatment, cell culture supernatants were collected and LDH activity was measured according to the manufacturer’s instructions. Absorbance was read at 490 nm using a SpectraMax Plus 384 microplate reader (Molecular Devices, LLC, San Jose, CA, USA). The percentage of cytotoxicity was calculated as [Cytotoxicity (%) = (Treated Sample LDH Release/Untreated Control LDH Release) × 100].

### Cell cycle analysis

Subsequently, we analyzed the cell cycle distribution of samples using flow cytometry. To do so, cells were treated with different concentrations of SH003 (i.e., 0, 100, 200, or 500 μg/mL) for 0, 24, 48, and 72 h, after which cells were harvested and fixed in 70% ethanol at 4°C overnight. Fixed cells were then stained with propidium iodide solution containing RNase A (BD Pharmingen, 550825, Franklin Lakes, NJ, USA) before samples were analyzed using a FACSCalibur flow cytometer (BD Biosciences, San Jose, CA, USA). Finally, the percentage of cells in the sub-G_1_ phase was determined using CellQuest Pro software, version 5.1 (BD Biosciences, San Jose, CA, USA).

### Western blot analysis

Caspase activation and EMT marker expression were then evaluated by Western blotting. E-cadherin and N-cadherin are critical markers of EMT in cancer. Loss of E-cadherin is linked to increased invasiveness and poor prognosis in oral squamous cell carcinoma (OSCC), while elevated N-cadherin promotes migration and correlates with tumor aggressiveness. The switch from E-cadherin to N-cadherin indicates EMT and is associated with the metastatic potential of oral cancers [20,21]. To do so, cells were treated with SH003 for 0, 24, 48, and 72 h or with Cucurbitacin D (0.1 μM), Decursin (100 μM), Formononetin (100 μM), or Nodakenin (100 μM) for 24 h. After these treatments, cells were lysed using RIPA buffer (Biosesang, R2002, Incheon, Republic of Korea) supplemented with protease inhibitor cocktail tissue 2 (Quartett, Berlin, Germany) and phosphatase inhibitor cocktail 2 (Sigma, St. Louis, MO, USA). Next, the protein concentration was determined using a BCA protein assay kit (Pierce Biotechnology, 23227, Rockford, IL, USA). Equal samples of protein (30 μg) were separated via SDS-PAGE before being transferred to Polyvinylidene Fluoride (PVDF) membranes (Merck Millipore, IPVH00010, Burlington, MA, USA). These membranes were then blocked with 5% nonfat milk in Phosphate-Buffered Saline (Dreamcell, P-2023-2, Seoul, Republic of Korea) with 0.1% tween-20 (Dreamcell, T-9001-1) (PBST) for one hour at room temperature, before being incubated overnight at 4°C with primary antibodies against caspase-3 (1:1000; Cell Signaling Technology, 9665, Danvers, MA, USA), 7 (1:1000; 12827), 9 (1:1000; Cell Signaling Technology, 9508), PARP (1:1000; Cell Signaling Technology, 9542), E-cadherin (1:2000; Cell Signaling Technology, 3195), N-cadherin (1:1000; Cell Signaling Technology, 13116), and GAPDH (1:10,000; Cell Signaling Technology, 5175T) (Cell Signaling Technology, Danvers, MA, USA). GAPDH was used as a loading control in Western blotting due to its stable and ubiquitous expression, ensuring accurate normalization of protein levels. We normalized the intensity of target protein bands to GAPDH to account for variations in protein loading, enhancing the reliability of our results. After washing with PBST, membranes were then incubated with anti-rabbit IgG (1:10,000–50,000; Cell Signaling Technology, 7074) or anti- mouse (1:20,000; Cell Signaling Technology, 7076) IgG HRP-conjugated secondary antibodies for another 1 h period at room temperature. Finally, protein bands were visualized using an EZ-Western detection kit (DoGen, DG-W250, Seoul, Republic of Korea) and recorded using a ChemiDoc™ MP System (Bio-Rad, Hercules, CA, USA). Protein abundance was then quantified using NIH ImageJ (National Institutes of Health, Bethesda, MD, USA).

### Wound healing assays

Wound healing assays were performed to evaluate cell migration by creating a gap in a cell monolayer and observing its closure over time. This method is a key metric for assessing metastatic potential, as enhanced migration indicates the ability of cancer cells to invade and spread. For these assays, 5 × 10^5^ cells were seeded in 12-well plates and grown to 90–100 confluence. A sterile pipette tip was used to create a scratch in the cell monolayer, and detached cells were removed by washing with PBS. The remaining adherent cells were treated with SH003 (500 μg/mL), Cucurbitacin D (0.1 μM), Decursin (100 μM), Formononetin (100 μM), or Nodakenin (100 μM). Next, images of the wound area were captured at 0, 6, 8, 13, 20, and 22 h using an Olympus CKX53 inverted microscope (Olympus Corporation, Shinjuku, Tokyo, Japan). The degree of wound closure could be determined by comparing the remaining wound area at each time point to the initial wound area. The number of migrating cells was quantified using NIH ImageJ software (National Institutes of Health, Bethesda, MD, USA). These specific time points were chosen based on preliminary experiments that indicated significant migration activity within this time frame for the selected cell lines.

### qRT-PCR of EMT markers

Quantitative reverse transcription PCR (qRT-PCR) was used to measure the expression of E- and N-cadherin. To do so, total RNA was first extracted from treated cells using a RNA-spin Total RNA Extraction Kit (Intron Biotechnology, 17211, Seongnam, Republic of Korea), and cDNA was reverse transcribed using a High Capacity cDNA Reverse Transciption Kit (Applied Biosystems, 4368814, Foster City, CA, USA). Next, qRT-PCR was performed using GoTaq^®^ qPCR Master Mix (Promega, A6001, Madison, WI, USA) on a qPCR QuantStudio 7 Flex Real-Time PCR platform (Applied Biosystems, Waltham, MA, USA). The following reaction conditions were used for all qPCR experiments: 95°C for 2 min for hot-start polymerase activation, followed by 40 cycles of denaturation at 95°C for 15 s, annealing and extension at 60°C for 1 min, and a final cooling step at 4°C. All primers used (synthesized by Cosmo Genetech Co. Ltd., Seoul, Republic of Korea) were as follows: Human E-cadherin, forward, 5′-CAGAAAGTTTTCCACCAAAG-3′, reverse, 5′-AAATGTGAGCAATTCTGCTT-3′; Human N-cadherin, forward, 5′-GCCCCTCAAGTGTTACCTCAA-3′, reverse, 5′-AGCCGAGTGATGGTCCAATTT-3′; Human GAPDH, forward, 5′-AATCCCATCACCATCTTCCA-3′, reverse 5′-TGGACTCCACGACGTACTCA-3′. The relative expression levels of E-cadherin and N-cadherin as determined by RT-qPCR were normalized to GAPDH (a house keeping gene) and their expression increase or decrease was calculated using the 2^−ΔΔCt^ method.

### Statistical analysis

All experiments were performed in triplicate to ensure reproducibility and reliability of the results, with data reported as mean ± standard deviation (SD). The statistical significance between group means was determined using two-tailed, two-sample Student’s *t*-tests with unequal variances in Microsoft Excel (Microsoft Corporation, Redmond, WA, USA) for multiple comparisons. *p*-values less than 0.05 (*p* < 0.05) were considered statistically significant. This approach ensures accurate quantification of variability and assessment of observed differences.

## Results

### SH003 significantly reduces cell viability but has a limited effect on rates of cell death

**Figure 1:**
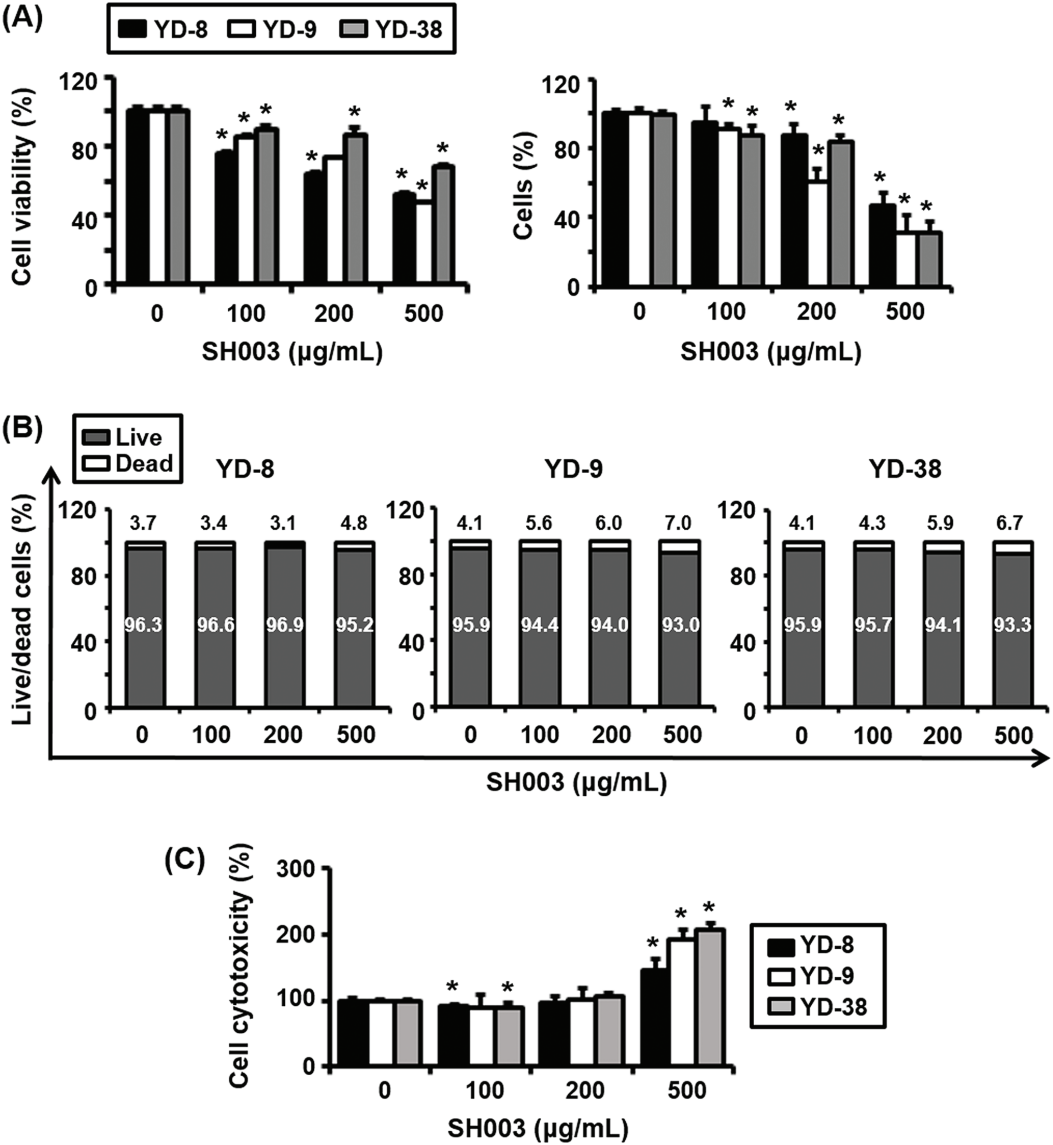
Effect of SH003 on cell viability and cytotoxicity in the YD-8, YD-9, and YD-38 oral cancer cell lines. (A) Left panel: cell viability of YD-8, YD-9, and YD-38 cells treated with increasing concentrations of SH003 (i.e., 0, 100, 200, 500 µg/mL) for 72 h. Viability was assessed using an MTT assay, and results are expressed as a percentage relative to untreated control cells. Right panel: Trypan Blue exclusion assay results showing reduced viable cell counts of YD-8, YD-9, and YD-38 cells treated with SH003 for 72 h, confirming dose-dependent cytotoxicity. (B) The proportion of living and dead cells following treatment with SH003, as assessed using live/dead cell assays, of YD-8, YD-9, and YD-38 cells, respectively. The data indicate a minimal increase in dead cells, even at 500 µg/mL, suggesting that SH003 reduces cell viability primarily through mechanisms other than direct cell death. (C) Cell cytotoxicity, as measured by LDH release after SH003 treatment for 15 h of YD-8, YD-9, and YD-38 cells. Data shown represent the mean ± SD of three independent experiments. *p < 0.05 compared to the untreated control. These results collectively demonstrate that SH003 decreases cell viability in a dose-dependent manner, with YD-8 and YD-9 cells exhibiting greater sensitivity to treatment than YD-38 cells.

Fig. 1 shows the dose-dependent effects of SH003 on cell viability and cytotoxicity against the YD-8, YD-9, and YD-38 cell lines. First, MTT assay results (Fig. 1A, left panel) show a significant, dose-dependent decrease in cell viability with increasing concentrations of SH003 (i.e., 0, 100, 200, and 500 μg/mL). Notably, YD-8 and YD-9 cells exhibited greater sensitivity to treatment with SH003 relative to YD-38 cells, as evidenced by a more pronounced reduction in cell viability at equivalent doses. Observed reductions in cell viability were corroborated by the results of Trypan Blue exclusion assays (Fig. 1A, right panel), which showed a corresponding decrease in viable cell number, suggesting increased cytotoxicity at higher concentrations of SH003.

In contrast, we also observed that live/dead cell assay results (Fig. 1B) showed that the proportion of dead cells increased by less than 10% even at the highest concentration of SH003 (i.e., 500 μg/mL). This suggests that while SH003 induces some level of cell death, this effect is relatively modest, and does not trigger cell death to a substantial degree. Next, the results of an LDH release assay (Fig. 1C) demonstrated significant membrane damage at higher concentrations of SH003, as evidenced by increased LDH release. This finding suggests that SH003 induces cytotoxic effects, yet the limited increase in dead cells observed in the live/dead assay indicates that SH003 may primarily affect cell viability through mechanisms other than inducing cell death. Notably, YD-8 and YD-9 cells exhibited greater susceptibility to SH003 compared to YD-38 cells.

**Figure 2:**
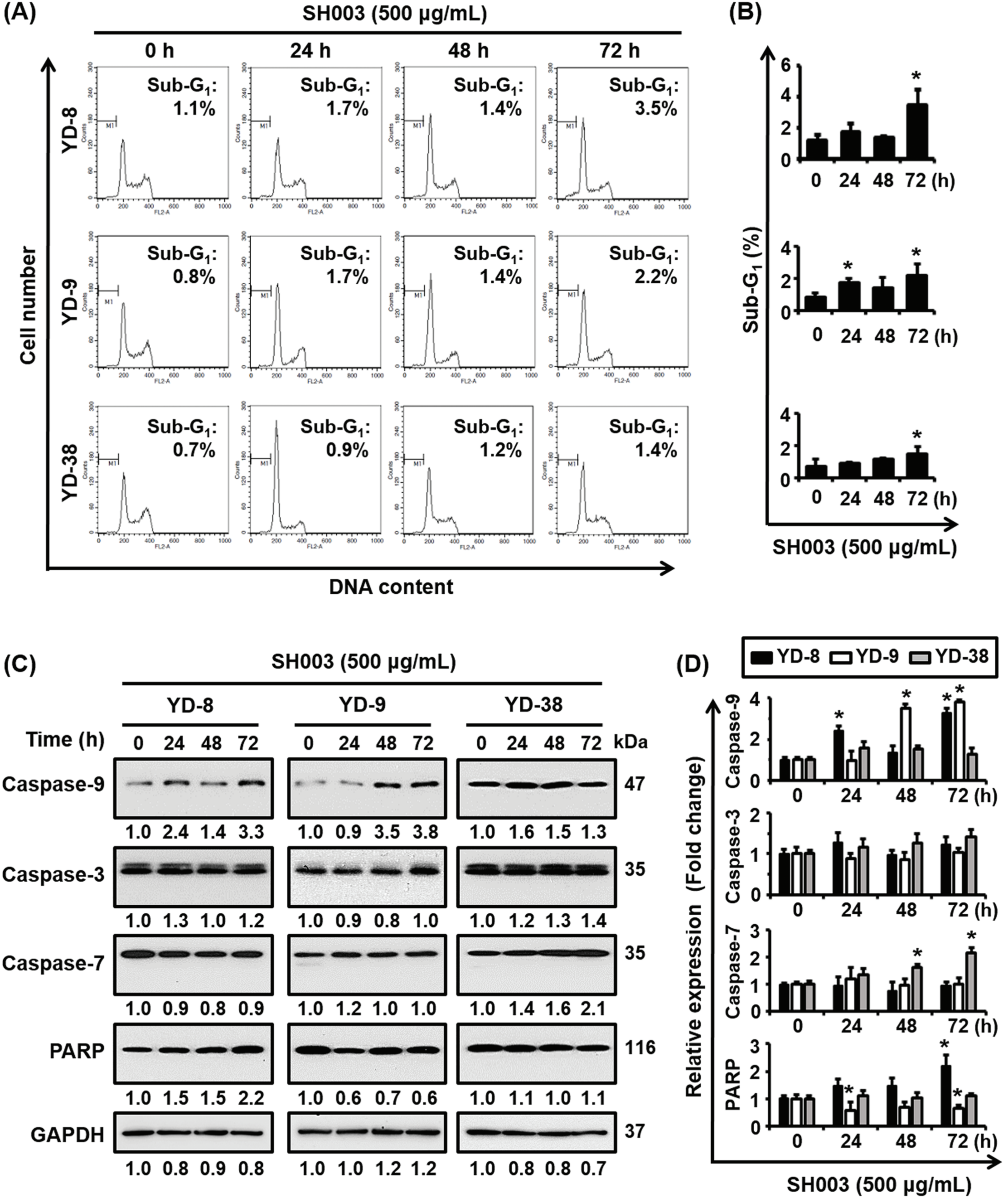
Impact of SH003 on the cell cycle and apoptosis induction in YD-8, YD-9, and YD-38 cell lines. (A) Cell cycle analysis of YD-8, YD-9, and YD-38 cells treated with SH003 (500 µg/mL) for 0, 24, 48, and 72 h. Flow cytometry was used to determine the percentage of cells in the sub-G_1_ phase. Representative histograms are shown for each cell line at different time points. (B) Quantification of the sub-G_1_ population from flow cytometry data. Results are presented as the percentage of sub-G_1_ cells at each time point (mean ± standard deviation; n = 3). (C) Western blot analysis showing the expression levels of apoptosis-related proteins (Caspase-9, Caspase-3, Caspase-7, and PARP) in YD-8, YD-9, and YD-38 cells treated with SH003 (500 µg/mL) for the indicated times. GAPDH was used as a loading control. (D) Quantification of Western blot results for apoptotic markers, showing fold changes relative to untreated controls (0 h). Data shown represent the results of three independent experiments. *p < 0.05 compared to an untreated control.

Fig. 2 examines the effects of SH003 on the cell cycle and the potential induction of apoptosis in YD-8, YD-9, and YD-38 cell lines. The analysis of the sub-G_1_ cell cycle phase is crucial for understanding SH003’s apoptotic effects in oral cancer cells, as this phase indicates DNA fragmentation, a key sign of apoptosis. By examining sub-G_1_ populations, we can determine if SH003 induces apoptosis and how its effects might differ across cell types. Here, cell cycle analysis results (Fig. 2A,B) show that there are minimal changes in the sub-G_1_ phase from 0 to 24, 48, and 72 h, which suggests that the SH003 treatment did not significantly induce apoptosis in any of the cell lines. Moreover, the percentage of cells in the sub-G_1_ phase remains relatively low, and showed only a slight increase over time. In addition, a further analysis of caspase activation (Fig. 2C,D) showed minimal activation of caspases-3, -7, and -9, as well as limited PARP cleavage over the same time period. The lack of significant caspase activation corroborates these cell cycle findings, thereby indicating that SH003 did not substantially induce apoptosis in these cell lines.

Taken together, these results collectively suggest that SH003 may reduce cell viability via mechanisms other than classical apoptosis, and may therefore involve nonapoptotic pathways or sublethal cellular stress responses.

### SH003 inhibits cell migration and modulates E-cadherin and N-cadherin expression in the YD-8, YD-9, and YD-38 cell lines

**Figure 3:**
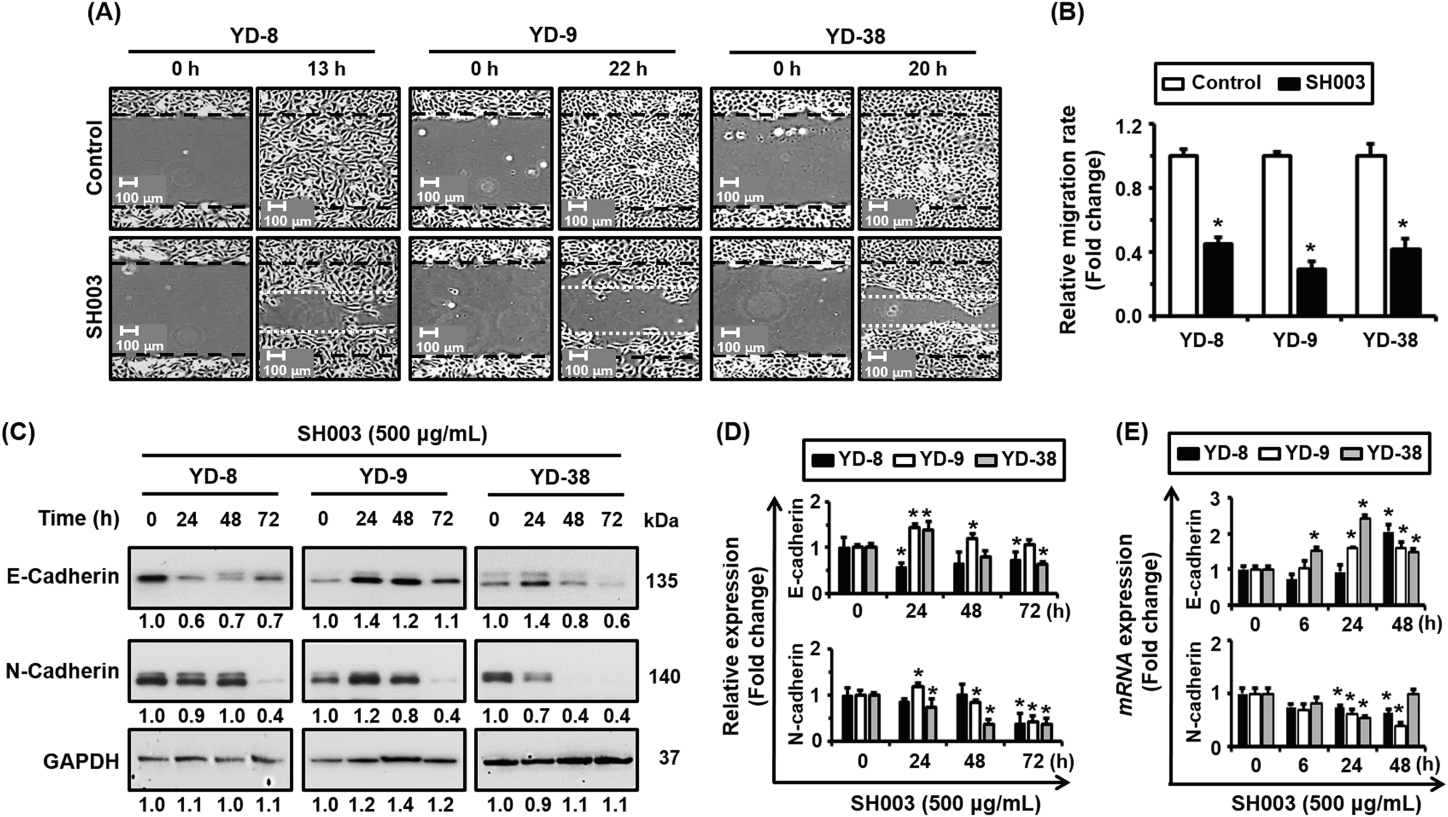
Inhibition of cell migration and EMT marker modulation following SH003 treatment in YD-8, YD-9, and YD-38 cell lines. (A) Wound healing assay showing reduced cell migration in YD-8, YD-9, and YD-38 cells treated with SH003 (500 µg/mL). Images were captured at 0 h and the indicated time points, with representative images shown for each treatment condition. (B) Quantification of wound healing assay results. The relative migration rate was measured using ImageJ software by analyzing the number of cells that migrated into the wound area. Results are expressed as fold change in migration relative to untreated control cells. White bars represent control groups, while black bars represent SH003-treated groups. (C) Western blot analysis of EMT markers (E-cadherin and N-cadherin protein levels) in YD-8, YD-9, and YD-38 cells following treatment with SH003 (500 µg/mL) for the indicated times. GAPDH was used as a loading control to normalize protein expression levels. (D) Quantification of Western blot results showing relative expression levels of EMT marker proteins. Data are presented as fold change relative to untreated control (0 h). (E) qRT-PCR analysis of EMT markers shows increased E-cadherin and decreased N-cadherin mRNA expression levels in YD-8, YD-9, and YD-38 cells treated with SH003 (500 µg/mL) for the indicated times. Results are normalized to GAPDH and expressed as fold change relative to the control group. All data represent the mean ± SD of three independent experiments. *p < 0.05 compared to the untreated control.

Fig. 3A,B shows the effects of SH003 on cell migration in the YD-8, YD-9, and YD-38 cell lines as revealed by a wound healing assay. For this experiment, cells were treated with SH003 at a concentration of 500 μg/mL, and their migration was observed at various time points. The untreated control groups exhibited substantial wound closure over time (i.e., by 13 h for YD-8, 22 h for YD-9, and 20 h for YD-38), thereby indicating active cell migration. In contrast, SH003-treated groups showed marked reductions in wound closure, suggesting that SH003 significantly inhibited cell migration in all three cell lines. This inhibition of migration supports SH003’s potential in preventing metastasis by impairing the movement of cancer cells.

Fig. 3C,D presents the results of an Western blot analysis that quantified the expression levels of E-cadherin and N-cadherin, two key markers of EMT, in YD-8, YD-9, and YD-38cells after treatment with SH003 over 0, 24, 48, and 72 h. These data indicated that SH003 treatment led to a time-dependent increase in E-cadherin expression in YD-9 cells, especially by 24 and 48 h, suggesting a potential shift toward an epithelial phenotype. Conversely, N-cadherin expression decreased in a time-dependent manner across all cell lines, with the most pronounced reduction observed in YD-9 and YD-38 cells. The modulation of cadherin expression indicates that SH003 may inhibit EMT, and may therefore reduce the migratory and invasive potential of these oral cancer cells.

Next, Fig. 3E shows the quantified mRNA expression levels of E-cadherin and N-cadherin in YD-8, YD-9, and YD-38 cells following SH003 (500 μg/mL) treatment at different time points (i.e., 0, 6, 24, or 48 h). Thus, we see a significant upregulation of E-cadherin mRNA at 24 and 48 h in all cell lines, with YD-9 showing the highest fold change. Conversely, N-cadherin mRNA expression was significantly downregulated following SH003 treatment, which is consistent with our Western blot findings. Taken together, these results suggest that SH003 inhibits cell migration and modulates key markers of EMT, such as E-cadherin and N-cadherin, in the YD-8, YD-9, and YD-38 oral cancer cell lines. The increased expression of E-cadherin and decreased expression of N-cadherin following SH003 treatment indicate a potential reversal of EMT, which may contribute to the observed reduced migratory capacity of these cells.

### Identification of active compounds present in SH003 that inhibit cell viability and migration

**Figure 4:**
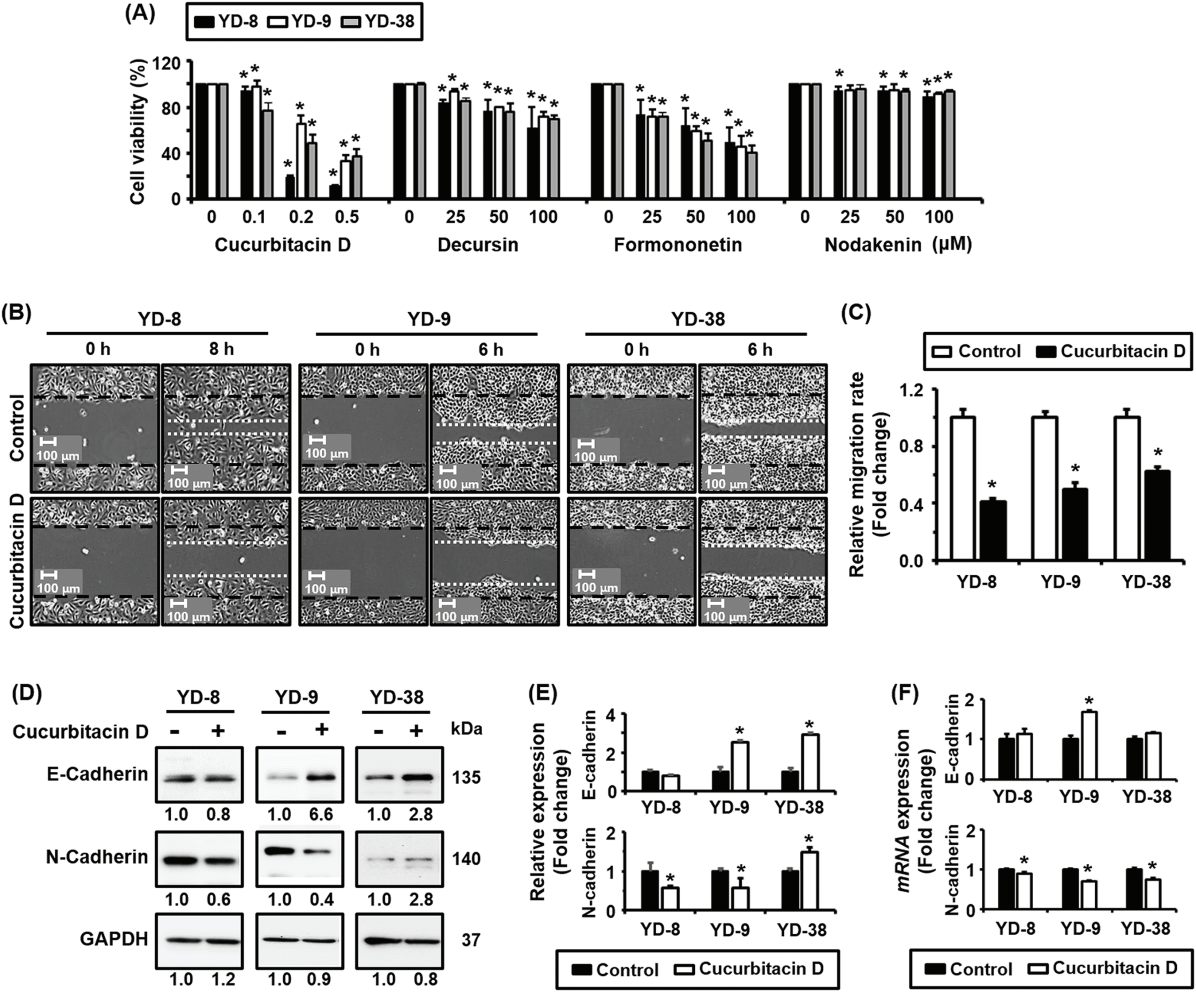
Identification of key active compounds in SH003 contributing to its effects on cell viability, migration inhibition, and EMT marker modulation. (A) MTT assay results showing the effects of various concentrations of Cucurbitacin D, Decursin, Formononetin, and Nodakenin on the viability of YD-8, YD-9, and YD-38 cells after 72 h of treatment. Curcurbitacin D exhibits the most potent inhibitory effect on cell viability across all tested cell lines. (B) Wound healing assay results showing the superior inhibitory effect of Cucurbitacin D (0.1 µM) on the migration of YD-8, YD-9, and YD-38 cells. Migration was assessed at 0 h and 6 or 8 h post-treatment, as indicated. Representative images highlight reduced wound closure in Cucurbitacin D-treated cells compared to untreated controls. (C) Quantification of wound healing assay results. The relative migration rate was measured using ImageJ software by analyzing the number of cells that migrated into the wound area. Data are expressed as fold change in migration relative to untreated control cells. White bars represent control groups, while black bars represent Cucurbitacin D-treated groups. (D) Western blot analysis of EMT-related protein levels, showing increased E-cadherin and decreased N-cadherin expression in YD-8, YD-9, and YD-38 cells treated with Cucurbitacin D (0.1 µM) for 24 h. GAPDH was used as a loading control. (E) Quantification of Western blot results showing relative expression levels of EMT marker proteins. Data are presented as fold change relative to untreated control. (F) qRT-PCR analysis confirming the upregulation of E-cadherin and downregulation of N-cadherin mRNA expression following treatment with Cucurbitacin D (0.1 µM) for 24 h, particularly in YD-9 cells. Data are normalized to GAPDH expression and presented as fold change relative to untreated controls. Data represent the mean ± SD of three independent experiments. *p < 0.05 compared to untreated control.

Given the activity of SH003, we then attempted to determine which compounds contribute most significantly to its observed effects on cell viability and migration. To do so, we tested several key constituents independently on the YD-8, YD-9, and YD-38 cell lines. Fig. 4A shows the effects of different SH003 compounds, including Cucurbitacin D, Decursin, Formononetin, and Nodakenin, on cell viability. We found that both Cucurbitacin D and Formononetin resulted in a significant dose-dependent reduction in cell viability, with Cucurbitacin D being the most potent, especially at lower concentrations (i.e., 0.1 to 0.5 μM). Furthermore, while both Decursin and Nodakenin reduced cell viability, their effects were more modest than Cucurbitacin D or Formononetin.

**Figure A1:**
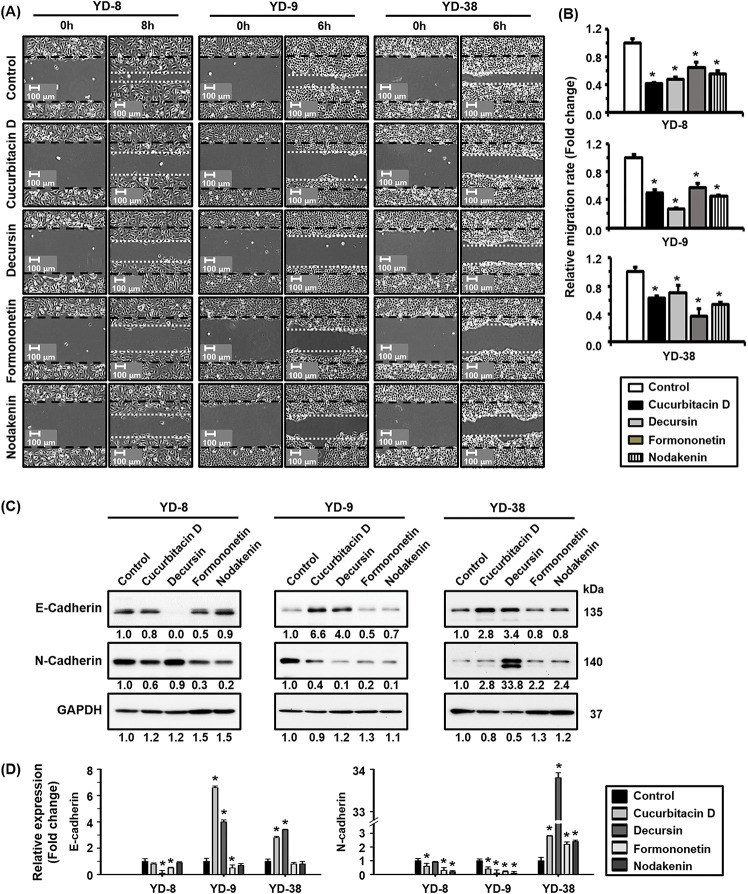
Identification of key active compounds in SH003 contributing to its effects on cell migration inhibition. (A) Wound healing assay showing the migration of YD-8, YD-9, and YD-38 cells treated with Cucurbitacin D (0.1 µM), Decursin (100 µM), Formononetin (100 µM), or Nodakenin (100 µM) at 0 h and 6 or 8 h post-treatment, as indicated. Representative images are shown for each treatment condition. (B) Quantification of wound healing assay results. The relative migration rate was measured using ImageJ software by analyzing the number of cells that migrated into the wound area. Data are presented as fold change in migration relative to untreated control cells. (C) Western blot analysis of EMT-related protein levels, including E-cadherin and N-cadherin, in YD-8, YD-9, and YD-38 cells treated with Cucurbitacin D (0.1 µM), Decursin (100 µM), Formononetin (100 µM), or Nodakenin (100 µM) for 24 h. GAPDH was used as a loading control. (D) Quantification of Western blot results showing relative expression levels of EMT marker proteins. Data are presented as fold change relative to untreated control. Data represent the mean ± SD of three independent experiments. *p < 0.05 compared to untreated control.

Next, Fig. 4B,C presents wound healing assay results for YD-8, YD-9, and YD-38 cell lines treated with Cucurbitacin D (0.1 μM), Decursin (100 μM), Formononetin (100 μM), and Nodakenin (100 μM). These results demonstrated that all four compounds significantly inhibited cell migration, as evidenced by reduced wound closure. Moreover, although Cucurbitacin D showed the most potent inhibitory effects on migration, Decursin, Formononetin, and Nodakenin also notably reduced cell migration, indicating that all of these compounds collectively contribute to the antimigratory effects of SH003. Data for Decursin, Formononetin, and Nodakenin can be found in Fig. A1A,B.

Fig. 4D,E presents a Western blot analysis of E-cadherin and N-cadherin expression following treatment with the same SH003 components. The treatment concentrations used in Fig. 4D are identical to those used in Fig. 4B, with Cucurbitacin D at 0.1 μM and Decursin, Formenonetin, and Nodakenin at 100 μM each. Of the tested compounds, Cucurbitacin D exerted the most statistically significant effect, and showed both the greatest increase in E-cadherin expression and decrease in N-cadherin expression. Based on these results, Cucurbitacin D is considered the major active compound present in SH003. Data for other compounds are shown in Fig. A1C,D.

To further confirm its role as the primary active compound, we specifically examined gene expression following treatment with Cucurbitacin D alone. Fig. 4F shows quantified mRNA expression levels of E-cadherin and N-cadherin following treatment with Cucurbitacin D, and confirms our earlier finding this compound plays a prominent role in modulating the expression of EMT- related markers. These results reveal a significant upregulation of E-cadherin and downregulation of N-cadherin mRNA, especially in YD-9 cells. By restoring E-cadherin expression and suppressing N-cadherin, Cucurbitacin D effectively reverses the EMT process, which is closely associated with the suppression of cancer cell migration.

Overall, these results suggest that among the compounds tested, Cucurbitacin D is likely the primary active agent in SH003 responsible for reducing cell viability and inhibiting migration, and primarily acts via exerting effects on the expression of EMT markers such as E-cadherin and N-cadherin. Together, these findings underscore why Cucurbitacin D is considered the main active compound in SH003, acting as a key regulator of EMT to reduce both cancer cell migration and viability.

## Discussion

In this study, we investigated the anticancer effects of SH003, traditional herbal formulation, and its major components (Cucurbitacin D, Decursin, Formononetin, and Nodakenin) on OSCC cell lines (YD-8, YD-9, and YD-38). Our results demonstrated that SH003 significantly reduced cell viability and inhibited cell migration, with YD-8 and YD-9 cells showing greater sensitivity than YD-38 cells.

While this study primarily utilized the MTT assay to assess cell viability, additional methods, such as ATP-based assays or real-time imaging, could provide complementary insights into the effects of SH003. For example, the study “*Effect of local anesthetics on cancer cells” (2020)* highlights alternative approaches that could be used to validate our results and further confirm SH003’s cytotoxic effects [22].

Interestingly, SH003 did not significantly induce apoptosis, as evidenced by minimal changes in sub-G_1_ phase cells and caspase activation. This suggests that its cytotoxic effects are mediated via alterative mechanisms potentially involving autophagy, senescence, or cell cycle arrest. The limited induction of apoptosis aligns with findings from other natural compounds, which often exert anticancer effects through diverse pathways, warranting further investigation into SH003’s precise mechanisms autophagy or necroptosis [23,24].

Among the individual components of SH003, Cucurbitacin D emerged as the most potent, demonstrating dose-dependent reductions in cell viability and significant inhibition of cell migration across all three cell lines. In addition, Cucurbitacin D modulated EMT markers by upregulating E-cadherin and downregulating N-cadherin which are critical regulators of tumor metastasis [25]. These findings are consistent with similar studies on natural compounds such as resveratrol and curcumin, which inhibit EMT by targeting key pathways like TGF-β and Wnt/β-catenin signaling [26,27]. EMT plays a pivotal role not only in cancer metastasis but also in drug resistance, as highlighted in *Cancer stem cells, epithelial-mesenchymal transition, ATP, and their roles in drug resistance in cancer, 2021* [28]. Thus, Targeting EMT-related pathways could enhance the effectiveness of SH003 against OSCC.

SH003’s inhibition of EMT and its potential to prevent metastasis make it a promising therapeutic agent [29]. EMT markers, such as E-cadherin and N-cadherin, have also been explored as prognostic biomarkers in cancers, including hepatocellular carcinoma [30]. These findings suggest that SH003’s ability to reverse EMT may have broader clinical applications in both treatment and prognosis.

Despite these promising results, the study is limited to *in vitro* experiments. Future studies should validate SH003’s efficacy *in vivo* models and investigate its mechanisms in more physiologically relevant systems, such as 3D culture models that mimic the tumor microenvironment [31,32]. Comparing SH003’s effects with those of similar natural compounds, such as berberine and baicalein, could also provide context for its efficacy and mechanism [33,34].

In addition to its potential as a monotherapy, SH003’s ability to modulate EMT and inhibit migration suggests its use as an adjunct to conventional therapies. By combining SH003 with chemotherapy or radiation, it may be possible to enhance therapeutic efficacy while reducing the side effects often associated with these treatments.

## Conclusion

SH003 demonstrates significant anticancer effects on OSCC cells, especially Cucurbitacin D. This study reveals that SH003 effectively reduced cell viability and inhibited cell migration in the YD-8, YD-9, and YD-38 cell lines. Notably, YD-8 and YD-9 cells showed slightly greater sensitivity to SH003 treatment, particularly in cell viability assays, while all three cell lines demonstrated significant reduction in migration capacity. This difference in sensitivity may be attributed to variations in baseline EMT marker expression or other molecular and genetic characteristics that make these cell lines more responsive to SH003’s mechanisms of action. Cucurbitacin D, the most potent component, plays a pivotal role in reversing EMT by upregulation E-cadherin and downregulation N-cadherin, thereby suppressing cancer cell migration and metastatic potential.

The therapeutic potential of SH003 likely arises from the synergistic effects of its multiple components rather than from a single dominant compound. These findings provide a foundation for further exploration of SH003 as a potential therapeutic for OSCC. Future studies should include *in vivo* validation, advanced models such as 3D cultures, and investigations into its molecular mechanisms. Additionally, examining its synergistic potential with existing therapies could lead to the development of safer and more effective combination treatments.

By targeting multiple aspects of cancer progression, including EMT and migration, SH003 offers a promising, multi-faceted approach to oral cancer therapy. Its potential as a natural compound-based treatment represents an important step forward in the development of less toxic and more effective cancer therapies, with implications extending to other cancer types.

## Data Availability

The authors confirm that the data supporting the findings of this study are available within the article.
